# Comparison of the internal thoracic artery flow dissected by video endoscopy or conventional technique

**DOI:** 10.1590/ACB360803

**Published:** 2021-10-08

**Authors:** Marcelo Curcio Gib, Thamyres Zanirati, Pauline Simas, Orlando Carlos Belmonte Wender, Leandro Totti Cavazzola

**Affiliations:** 1 MD. Cardiovascular Surgery Department - Hospital de Clínicas de Porto Alegre (HCPA) – Porto Alegre (RS), Brazil.; 2 Graduate student. School of Medicine - Universidade Federal do Rio Grande do Sul (UFRGS) – Porto Alegre (RS), Brazil; 3 PhD, Full Professor. Surgery Department - Hospital de Clínicas de Porto Alegre (HCPA) – Porto Alegre (RS), Brazil.; 4 PhD, Associate Professor. Surgery Department - Hospital de Clínicas de Porto Alegre (HCPA) – Porto Alegre (RS), Brazil.

**Keywords:** Coronary Artery Bypass, Thoracic Surgery, Mammary Arteries, Models, Animal

## Abstract

**Purpose:**

To compare the blood flow in the internal thoracic artery when dissected endoscopically in a conventional manner, in addition to develop a reliable experimental training model for the surgical team.

**Methods:**

Paired experimental study. Ten pigs were operated and had both internal thoracic arteries dissected, the right with a conventional technique and the left by video endoscopy. The main outcomes to be studied were flow, length, and time of dissection of each vessel.

**Results:**

Blood flow measurements were performed with mean heart rate of 100 ± 16 bpm and mean arterial pressure of 89.7 ± 13 mm Hg. The mean blood flow of endoscopic dissection of the internal thoracic artery was 170.2 ± 66.3 mL/min and by direct view was 180.8 ± 70.5 (p = 0.26). Thus, there was no statistically significant difference between the flows, showing no inferiority between the methods.

**Conclusions:**

The minimally invasive dissection of the internal thoracic artery was shown to be not inferior to the dissection by open technique in relation to the blood flow in the present experimental model. In addition, the model that we replicated was shown to be adequate for the development of the learning curve and improvement of the endoscopic abilities.

## Introduction

The search for minimally invasive (MI) procedures, both by patients and by professionals, has been increasing nowadays. However, new technologies are usually accompanied by high cost, high learning curve, and doubtful results in the initial few cases^1^. Before these facts, it is becoming increasingly important to conduct training in experimental models and on simulators in search of optimized results since the beginning of clinical experience^2^.

Coronary artery bypass grafting (CABG) is a well-established procedure in medical practice and presents excellent results^2–4^. The proposal of new approaches, especially the MI ones with less surgical trauma and a lower rate of potential complications, necessarily requires the maintenance of its current results. One of the pillars of CABG success, which has kept it competitive against stents, is the use of the left internal thoracic artery (ITA)^5^^–^ 7. The aim of this study was to compare the blood flow of the left ITA, that is endoscopically dissected, to the right ITA blood flow, dissected in a conventional manner, in addition to develop a reliable experimental training model for the surgical team.

## Methods

The present study was approved by the Ethics Committee on the Use of Animals of the Hospital de Clínicas de Porto Alegre (no. 130518). Ten adult Landrace pigs weighing 30 to 40 kg were operated on. In all animals, the two ITAs were dissected in a skeletonized fashion, the left one using video endoscopy and the right one using a median sternotomy. The sample size was calculated for paired samples with a maximum difference of 20% for no inferiority based on Demertzis’ work^8^, with a minimum number of seven animals required. All animals were treated according to the Ethical Code for Animal Experimentation (ARRIVE guideline).

### Preparing the animals

The pigs were housed for acclimatization for at least 24 hours before the procedures. They were subjected to solids fasting for 18 hours and nothing by mouth for 12 hours. Standardized preanesthetic medication consisted of ketamine hydrochloride, 15 mg/kg; meperidine, 5 mg/kg; and midazolam, 0.8 mg/kg intramuscularly, in the lodging and without the need for mechanical containment. Peripheral vein puncture of one of the ears and monitoring was installed for electrocardiogram and pulse oximeter. Preoxygenation was performed for 5 minutes before anesthetic induction, performed with propofol 4 mg/kg and instillation of 2% lidocaine at a dose of 0.5 mL for reduction of laryngospasm. Endotracheal intubation was done with tube 7 or 7.5. Maintenance of general anesthesia was performed with intravenous infusion of propofol at 0.8 mg/kg/minute and fentanyl 50 µg/kg.

The animal was initially positioned in the right lateral decubitus position with the hyperextended left upper limb exposing the axilla and the lower tip of the scapula during endoscopic video dissection of the left internal thoracic artery (LITA) after repositioning in open dorsal recumbency.

### Surgical instruments and positioning of the trocars

**Figure 1:**
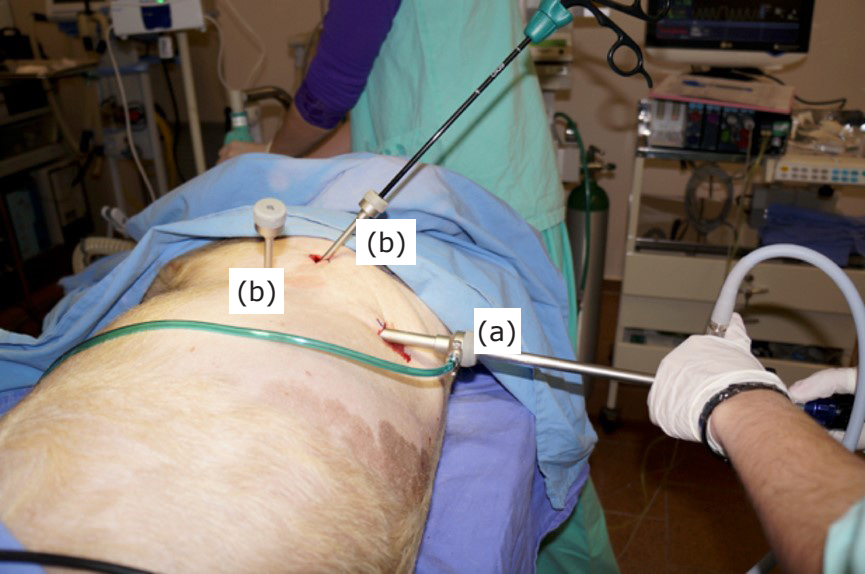
Positioning of the trocars. **(a)** Camera port.**(b)** Port for the dissection and coagulation device.

For all experiments, the same surgical material was used. In the video that shows endoscopy, a standard 30-mm angled optics, a video camera, two 5-mm trocars, a Storz^®^ surgery video tower, an angled dissecting forceps, an endoscopic hook attached to a monopolar electrocautery, straight traction forceps, and a 200-clamp clip were used. The experimental model developed by Demertzis^8^ was used for endoscopic approach. The portal of the camera was placed in the sixth intercostal space (ICS) left, 5 to 10 cm posterior to the posterior axillary line (PAL) and the working portals in the fifth and seventh ICS on the PAL. The left thoracic cavity was inflated by CO_2_, with a constant 4-8-mm Hg pressure. Once the thoracic cavity was accessed, the animals were manually ventilated until extraction of the trocars to avoid bradycardia and death (Fig. 1).

### Anatomy of the ITA swine

The porcine ITA is a direct branch of the subclavian artery, as in humans, and it originates at an angle of approximately 90°. It travels under the corresponding parietal pleura in the anterior mediastinum, being posterior to the costochondral cartilages. In its more cranial portion, the mammary was found devoid of branches and could be visualized through the endothoracic fascia. It has an approximate diameter of 5 to 6 mm, being accompanied by larger veins with diameters of 6 to 12 mm and extremely friable walls^8^. From 8 to 10 cm of its origin, a thick muscular layer covers the ITA with approximately 1 to 2 cm until its bifurcation in the final portion of the thorax a few centimeters before penetrating the diaphragm. In this portion, the ITA emits several branches that leave mainly the anterior surface of the vessel and some of them of great caliber.

### ITA endoscopic video approach

Using dissecting forceps, the ITA was approached by creating a window at the middle level of its more cranial portion. The artery was dissected initially in its cranial portion in which it presents, in most of the times, devoid of branches. Releasing the entire muscle portion completes the dissection. In this follow-up, the entire extension was first exposed, and the obliteration of each of its branches was performed–with clipping and/or cauterization according to the caliber of the branch.

### Open approach of ITA

In the supine position, a longitudinal thoracic incision was made on the midline, and total median sternotomy was performed. The anterior mediastinum was approached with the use of Finochietto retractor. Under direct view, the right ITA was skeletonized from its cranial portion to its distal bifurcation, using monopolar electrocautery, anatomical forceps, Metzenbaun and Potts scissors, as well as clipping or cauterization of its branches, following the criteria used on the contralateral side.

### Blood flow analysis and measurement of ITA length

At the end of complete dissection of both mammary arteries, intravenous heparin (1 mg/kg) was administered. The distal portion of each artery was clipped near the bifurcation and externalized medially on the pericardium. Each ITA was completely wrapped with gauze, and 10 mL of papaverine topical solution was instilled. A direct puncture in ascending aorta artery was performed to measure the mean arterial pressure (MAP). After another 5 minutes, the length of ITA was measured using a ruler, from its origin to the distal clip. A complete transverse arteriotomy was then performed at its distal portion to remove the clip, and blood flow was collected for 30 seconds in a vial and measured and multiplied by 2 to avoid hemodynamic deterioration. Saline solution was used to obtain a MAP and heart rate (HR) similar to the initial ones followed by measuring the flow in the contralateral ITA.

### Definition of surgical times

Zero time (ZT) was defined as the time when the animal was anesthetized and positioned for the procedure. The time interval between the beginning of the endoscopic dissection and the removal of the trocars was defined as endoscopic time (ET). Open time (OT) was defined as the time when cutaneous incision was started at the right ITA clipping.

### Statistical analysis

t-Tests for paired samples and Fisher’s exact test for categorical variables were used. Statistical analysis was performed by statistical program (StatGraphics Plus 2.1, Statistical Graphics Corporation, United States), consisting of comparative tests of the means (Student’s t-test) and analysis of variance (ANOVA). The results were represented as mean ± standard error of the mean, with a level of statistical significance defined for p < 0.05.

## Results

**Figure 2:**
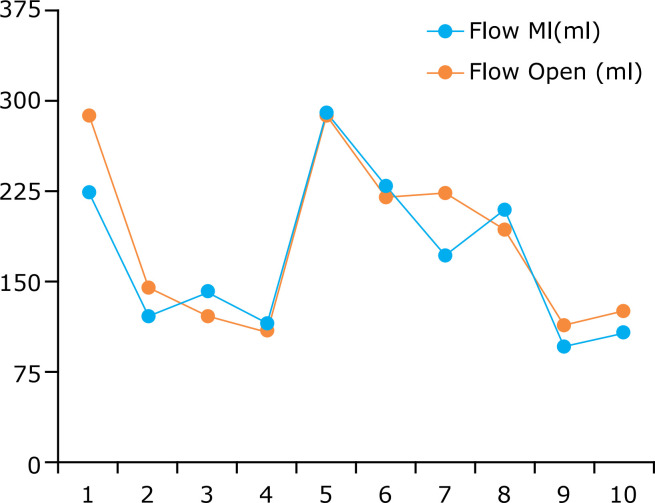
Mammary flow in milliliters per minute in each of the 10 studied pigs through free bleeding.

The same surgeon and team performed all surgeries. Ten pigs were operated on, and all mammary arteries were successfully dissected. The mean weight of the animals was 34.2 ± 2.21 kg (Table 1). Blood flow measurements were performed with an average HR of 100 ± 16 bpm, and MAP of 89.7 ± 13 mm Hg. The mean of the ITA blood flow was similar between the two groups. The mean blood flow in the endoscopic dissection was 170.2 ± 66.3 mL/min and by direct view was 180.8 ± 70.5 mL/min (p = 0.260; 95% confidence interval–IC95% -30.5–9.23) (Fig. 2). Thus, there was no statistically significant difference between the flows.

**Table 1:** Demographic data of animals.

Animal	Weight (kg)	MAP (mmHg)	HR (bpm)
**1**	34.3	90	98
**2**	34.6	86	92
**3**	34.2	80	85
**4**	34	110	100
**5**	31.2	95	87
**6**	31	93	87
**7**	33.2	60	91
**8**	38.3	90	120
**9**	36.7	97	135
**10**	35.1	96	106

MAP: main arterial pressure; HR: heart rate.

Mammary flow in milliliters per minute in each of the 10 pigs

**Figure 3:**
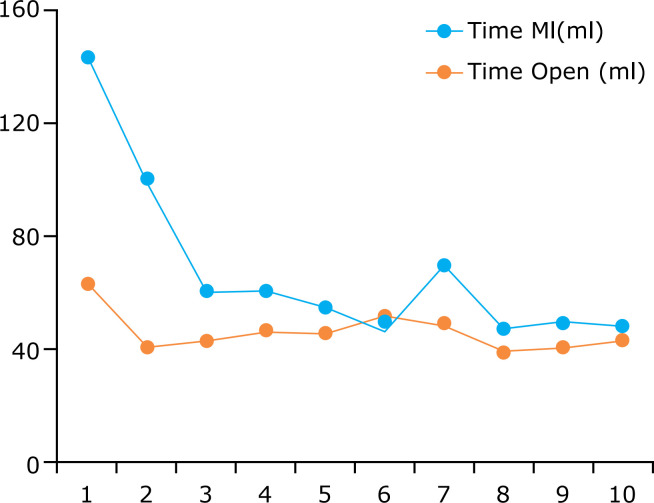
Comparison of the dissection time of the mammary arteries, in minutes, by endoscopic and direct vision.

Following the findings already described in the literature, the time taken for the endoscopic procedure was longer than that of the direct view procedure with a median of 57.5 (49.5–77.5 min) and 43.5 min (39.75–48.5 min), respectively, with statistical difference (p = 0.008) (Fig. 3).

Time to complete each dissection in minutes

**Figure 4:**
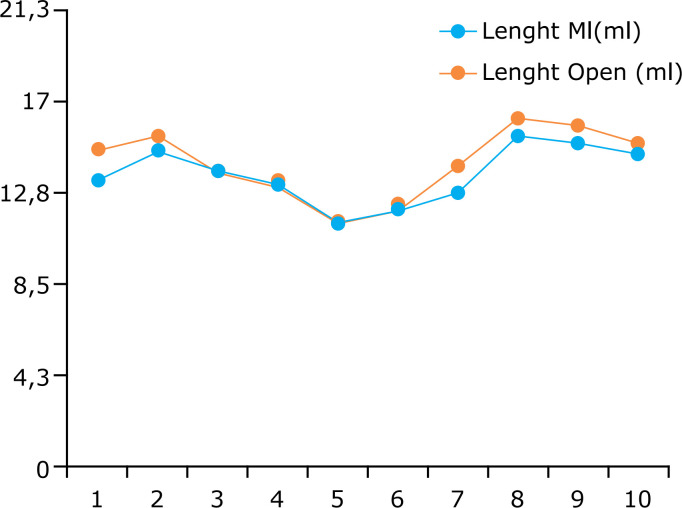
Comparison of internal thoracic artery length in centimeters after preparation with papaverine solution and warm saline solution.

A learning curve with a significant improvement in the time after case 3 was observed. An oscillation occurred in case 7, which presented a large inflammatory process and parietal pleural adhesions, requiring a longer time for the release of the cavity and the artery, which had repercussion on the value of the endoscopic blood flow. The length of the open-dissected mammary was significantly higher, 13.73 ± 1.37 in the endoscopic group and 14.22 ± 1.58 in the open IC95% (-0.87–-0.10) (p = 0.018) (Fig. 4).

Length of mammary (centimeters) in each of 10 pigs

There were three cases of branch dissection without loss of ITA flow, one in the open technique and two in the endoscopic one, all related to the placement of the metal clips. In this series, no cases of major bleeding or transoperative deaths were observed. In three cases, cardiac arrhythmias were observed during the blood flow measurement phase, with two ventricular fibrillations requiring internal electrical cardioversion with 10 J and one sustained ventricular tachycardia with spontaneous reversal. The final cost of the project was US$ 2,117.65 (less than US$ 250 per case). This final computed cost is only for the pigs and disposable materials used in the experiment, and does not include facility or housing expenditures.

## Discussion

Myocardial revascularization (CABG) is one of the most frequent surgeries performed worldwide, and in the last three decades many advances have occurred. One of the fundamental pillars of the long-term success of this procedure is the choice of grafts^9^. The LITA is the major determinant of a good late outcome in CABG^10^. It is believed that this occurs because of the structure and function of the artery, in which the middle tunica receives blood flow from the lumen of the vessel itself^11^^,^ 12. The literature states that the mean patency rate of LITA at 10 and 15 years to be 93 and 88%, respectively, whereas saphenous vein patency in these same periods to be 71 and 32%^3^^,^ 4 ^,^ 6 ^,^ 10. This superiority in graft patency results in an increase in survival over 10 years (LITA for anterior descending artery–ADA 82.6%, saphenous for ADA 71%) with a lower incidence of myocardial infarction, hospitalization for cardiac events or reoperation^13^^–^ 15.

The current interest in being able to perform the procedures through increasingly smaller incisions has led to the introduction of assisted video thoracoscopy techniques, the field of cardiac surgery being the largest exponent of robotic surgery. MI surgery has proven to be beneficial to both medical and economical aspects and is already classified as a gold standard for many procedures in various specialties^16^^,^ 17.

Technological advances have brought new surgical instruments to the armamentarium of cardiac surgery, allowing the accelerated development of MI techniques^15^. Ideally, MI myocardial revascularization should attempt to include the following aspects: small access, with minimal rib spacing, performed without the use of extracorporeal circulation, offering complete revascularization, and if possible, arterial grafts^16^^,^ 18 ^,^ 19.

There is a clear learning curve to be followed by every surgeon who engages with MI techniques. This step varies for each procedure, causing fear in many professionals not to use a well-established technique and good results if risking a new approach^1^. Virtual reality and video training are used in a preclinical phase to reduce training time in the operating room^20^. Simulation offers significant benefits to surgical trainees by allowing for repeated practice of a specific skill set in a safe and controlled environment^21^. Experimental models can help surgeons become familiar with the procedure, consequently speeding up their learning process.

Endoscopic CABG has been developed for the last two decades by several large cardiac surgery centers. The incorporation of robotic technology allowed the progression of this procedure, making it reality^1^. Because it is an extremely complex and expensive procedure, robotic CABG (TECAB) requires a specific and gradual training of surgeons to master this approach and a highly powerful medical center for such a procedure. Valdis^2^ performed the first randomized clinical trial to compare the three different training modes for robotic cardiac surgery, and the found results highlight the beneficial use of wet labs in robotic simulation training, suggesting their use whenever possible for a fast and safe acquisition of robotic skills^2^.

A key step in the development TECAB is the dissection of ITA. The preparation of the ITA under direct vision through a small incision is feasible but requires significant rib spacing and an expressive distortion of the anterior thoracic wall^8^. In this scenario, endoscopic video dissection of the ITA appears as a prerequisite in performing a TECAB^19^. Endoscopic manipulation of the ITA in a closed thoracic cavity is a technically delicate procedure and is associated with a substantial risk of vessel damage^8^. There are limited possibilities of controlling heavy bleeding of the mammary or its branches. Consistent surgical skills are needed to reduce the number of ITA injuries or urgency conversions for the open technique.

The comparison of the ITA flow in our study showed no inferiority when compared with the traditional method encouraging us to move forward in this line of treatment. The experimental model used allows direct comparison of the two techniques regarding the blood flow in each animal, allowing the observation of errors, correctness, and repair of the technique case by case. The results suggest that we can reach outcomes similar to those of conventional surgery with the use of video endoscopy and allow the widespread use of hybrid procedures, besides training surgeons willing to initiate the use of MI techniques.

The assisted video procedure is technically demanding, even for surgeons with good thoracoscopic skills. The learning of these skills can be protracted, especially for those who are not familiar with video surgery in their everyday lives. An experimental training model can help surgeons become familiar with the procedure and thus speed up their learning process. In our study, this curve was more significant in the first three cases and reached stability between the fourth case and the tenth case. A significant difference between the times of dissection was observed and may be explained by the long experience in performing open dissections. However, a trend in time reduction may already be noted in Fig. 3. Likewise, it is believed that the graft length has been shown to be longer in the open mode, which may be because of the considerable caution the surgeon preferred in proceeding with the dissection in its caudal portion, under which the muscle layer is thicker, and the diameter of the vessel is finer.

Studies have shown that swine models are ideal for in*-*vivo training of MI surgical techniques^2^^,^ 18 ^,^ 22 ^–^ 24. A standardized experimental model in pigs represents a useful tool for the training of surgeons to develop their skills. The model developed by Jiga *et al*.^22^ seems to have a rapid learning curve, requiring minimal experience in laparoscopic surgery, serving as a practical preparation for performing more complex procedures, such as dissection of the ITA for MI cardiac surgery. However, this model has the disadvantage that the dissection of the ITA occurs only in the proximal third, a relatively small portion (mean of 3.2 cm in length), and is devoid of branches in swine.

A great advantage of the model adapted in the present study is the low cost associated with the simplicity of the surgical material required for the procedures^8^. This low cost also seems to serve as an inclusion factor for mid-sized services or from less developed countries to enter the world of MI procedures. The importance of the development of experimental models for the treatment of ischemic heart disease is growing, even as maintaining similar results to those already obtained by conventional surgery.

Concerning the limitations of the present study, it is important to consider some issues. There was a significant variation in the ease of access to the ITA region, shown to be proportional to the animal size. Pigs weighing close to 40 kg allowed a better manipulation of the instruments, as against in animals with less than 35 kg. The fragility of the ITA wall is a factor that must be observed, considering its dissection is extremely easy. Finally, the experience of the surgeon regarding the direct view surgical technique should be emphasized, which may have been biased when compared with surgical times and length.

Minimally invasive techniques are the future of cardiovascular surgical procedures. The importance of the development of training models that allow a broad improvement of the professionals in a financially accessible manner is increasing. Our study intends to stimulate cardiovascular surgeons to use new therapies for the treatment of ischemic heart disease with results similar to those already obtained by conventional surgery.

## Conclusions

The MI dissection of the ITA has shown to be not inferior to the dissection by open technique in the present experimental model. The model has shown to be adequate to develop and improve endoscopic skills.
